# Volatile profiles from over-ripe purée of Thai mango varieties and their physiochemical properties during heat processing

**DOI:** 10.1371/journal.pone.0248657

**Published:** 2021-03-17

**Authors:** Malaiporn Wongkaew, Jiraporn Sangta, Sunee Chansakaow, Kittisak Jantanasakulwong, Pornchai Rachtanapun, Sarana Rose Sommano

**Affiliations:** 1 Faculty of Agriculture, Department of Plant and Soil Sciences, Plant Bioactive Compound Laboratory, Chiang Mai University, Chiang Mai, Thailand; 2 Faculty of Integrated of Science and Technology, Programme of Food Production and Innovation, Rajamangala University of Technology Lanna, Chiang Mai, Thailand; 3 Major of Biotechnology, Graduate School, Chiang Mai University, Chiang Mai, Thailand; 4 Faculty of Pharmacy, Department of Pharmaceutical Sciences, Chiang Mai University, Chiang Mai, Thailand; 5 Cluster of Agro Bio-Circular-Green Industry (Agro BCG), Chiang Mai University, Chiang Mai, Thailand; 6 Faculty of Agro-Industry, School of Agro-Industry, Chiang Mai University, Chiang Mai, Thailand; Institute for Biological Research, SERBIA

## Abstract

This research investigated volatile profiles of over-ripe Thai mango purée during thermal processing by solid-phase extraction, volatile quantification by XAD-2-solvent extraction, as well as descriptive sensory analysis. Overripe fruits of three varieties were analyzed for the ripening stage using specific gravity as well as firmness and the physiochemical properties were also reported. We found that aromatic profiles could be used as true representative to describe Thai mango identities of each varieties. A simple and straightforward heat treatment had differing effects on aroma characteristics and those effects were dependent with mango varieties. Indeed, the amount of terpene hydrocarbons and oxygenated sesquiterpenoids alternated after heat treatment. All descriptive attributes of heated ‘sam-pee’ purée were intensified while, heat treatment significantly improved only “mango identity” in ‘maha-chanok’ and “fermented” odour in ‘keaw’ purée. With or without heat treatment, the volatile profiles of ‘maha-chanok’ remained quite stable while heating played a significant role on chemical ingredients of ‘keaw’ and ‘sam-pee’. Our study demonstrated that the manufacturing of the over-ripe mango into the products of high market value, selection of varieties is vitally important based upon their specific aroma characteristics before and after processing.

## Introduction

Mango is one of the world’s most traded tropical fruits with a global annual production of approximately 50 million metric tons between 2016–2018 [1–3]. In Thailand, mango production contributes substantially to the economics and Thai varieties also secure good market positions in both domestic and international market due to the uniqueness in taste and aroma [4]. For nutritional values, mango flesh contains fibres, minerals, vitamins and natural antioxidants [5]. It is also good sources of bioactive compounds such as beta-carotene, ascorbic acid and terpenoids [6,7]. The terpenoid hydrocarbons are partly responsible for the aroma and flavours of the fruits. By using metabolomics tools such as mass spectrometry-based study, more than 300 free volatiles have been identified and vary among varieties [8,9]. Monoterpenes and sesquiterpenes such as 3-carene and α-copaene are typical volatile compounds that embody the flavour and aroma of mangoes [10,11].

Excessive of the supply over the demand, may lead to a decrease in price and unsold produce. Therefore, over-ripe mangoes are usually shifted to food processing industries. Finished products include canned (49%) and dried (6%) mangoes which accounted for more than 50% of exported Thai mango products [4]. The most frequently used varieties for processing are ‘sampee’ and ‘keaw’ for candies, ‘maha- chanok’ for juice and ‘chok-anan’ for dried mango products by which heat treatment is commonly incorporated [12]. During processing, heating can lead to physiological and biochemical changes of the mango pulp including carotenoid degradation and volatile profile alterations [13,14] but has little effect on more stable compounds such as acids and sugars [15,16].

Mango aroma is an important quality attribute affecting consumer preference [17]. To this extend, Castro *et al*. [18] added that the volatile fraction of the fruit makes a major contribution to the consumer’s overall perception of the mango product. Thus, in order to improve the quality of the finished products or to satisfactory fulfill consumer’s perceptions of true aroma and flavour of particular varieties, it is significant to understand changes of aromatic profiles before and after heat processing. Therefore, the effects of heat processing on the aromatic profiles of three Thai mango varieties were conducted. The outcomes of these will possibly facilitate the optimisation of the treatments that minimise the loss of the key aromatic identities for product development of the higher affluent market.

## Material and methods

### Fruit samples

‘Sam-pee’, ‘maha-chanok’ and ‘keaw’ mangoes were harvested at a commercial ripening stage from the orchard of Maejo University in Sansai district, Chiang Mai, Thailand. Mango fruits were dipped in an aqueous solution of ethephon (Ethrel^®^) 48% w/v at 500 ppm for 5 min [19] and then ripened at ambient temperature (24 ± 1°C) till passing 2 days of the commercial ripening stage which is described as soft to touch with the appropriate skin colour for each of the given cultivars [12,20]. Fruits were used immediately for the analysis of volatile profiles or kept at -21°C for physiochemical analyses.

### Ripening stage determination

#### Specific gravity

Specific gravity determination was according to Archimedes’ theory after the methods of Khalid *et al*. [21] and Chen *et al*. [22].

#### Texture analysis

Texture analysis of mango was measured by compression on the surface of the fruits in different positions using Texture Analyzer (TAXT Plus, Stable Micro Systems, UK) attached with P36 cylinder stainless. The test speed was set at 1 mm/s in a distance of 7.5 mm. The maximum peak force was expressed as firmness (N) [23]. Ten fruits were used for each variety.

### Physicochemical analyses

Flesh of each variety was puréed for 1 min in an immersion blender at high speed (HR2068 Philips, Thailand) and further blended in a hand-held homogeniser (T25 Ultra-turrax, Germany) for 2 min at 20,000 rpm. Thereafter, the pH of the sample was measured using a digital pH meter (Ultra Basic Benchtop Meters, Model UB-10, Denver Instrument, USA). Also, the total titratable acidity (TA) was determined by titration of the mango purée with 0.1 N NaOH to a pH of 8.1 and expressed as g citric acid per 100 g purée. Total soluble solid (TSS) was evaluated using a digital hand-held pocket refractometer [Atago PAL-1 (3810), ATAGO Co., Ltd, Tokyo, Japan] and their concentrations were designated in percentage.

For sugar contents, the extraction method of Wang *et al*. [24] was followed with slight modification. Five grams of mango purée were homogenised with 25 mL extraction solution containing 3.2 g/L acetic acid and 2.4 g/L potassium ferrocyanide, dissolved in double distilled water. The extraction was done on ice and kept still for 20 min. The mixture was then centrifuged at 12,000 x g for 20 min at 4°C. The supernatant was collected and diluted to 150 mL with double distilled water. Prior to the HPLC analysis, the resulting extract was filtered through a 0.22 μm membrane filter. Separations were achieved on HPLC (Shimadzu LC-20A HPLC, Shimadzu, Japan) using NH_2_ 5 μm, 250×4.6 mm I.D column with acetonitrile:water (75:25) as mobile phase. The flow rate was 1 mL/min, and the column temperature was 40°C. Separated sugars were detected using RID detector (cell temperature = 40°C, response = 3 and AUX range = 10^3^). Fructose, glucose, and sucrose were identified by the retention of time and quantified using the standard curves of the individual sugars. Maltose at a concentration of 0.1 M was used as internal standard.

### Heat treatment of the purée

The above purée was then heated with constant stirring until the temperature reached 85°C for 15 min. After heating, the purée was cooled immediately to 35°C, placed in a plastic Ziploc bag, sealed, and kept at -18°C until used for analysis.

### Descriptive attribute determination and sensory analysis

Mango volatiles were analysed using automated Solid Phase Microextraction (SPME) after the methods of Beaulieu and Lea [25] and Wen *et al*. [26] with some adaptions. The homogenised purée (3 mL without foam) was pipetted into 10 mL glass vials containing 1.1 g NaCl which were sealed with a steel crimp cap fitted with a Teflon/silicon septum and heated at 40°C. After incubation to allow an appropriate equilibrium time, 4 hr, the SPME holder coated with 100 μm polydimethylsiloxane (PDMS) was inserted into the chamber and the adsorption time was 15 min. Thereafter, it was thermally desorbed in the S/SL injector for 10 min at 250°C for 1 min in the injection port of a Gas Chromatography (GC), equipped with a DB-5ms column and HP 5973 mass-selective detector with a split ratio of 1:10. Injector and transfer line temperatures were 230 and 250°C, respectively. The oven temperature was programed at 40°C for 3 min, then 190°C at 3°C/min and 240°C at 8°C/min for 5 min, with helium as carrier gas (1.1 mL/min). Mass spectra was taken at 70 eV, which was identified by comparison with NIST/EPA/NIH Mass Spectral Library (NIST 05) library. The identified compounds were then classified according to their characteristic sensory attributes which were used to develop the descriptions of the aroma attributes during training in the following sensory session.

Ten accessors (4 males and 6 females) age ranged from 21–40 years old evaluated the fresh and heated purée prepared from each of the three varieties. Training was intensively conducted in a 3 session per week for 4 weeks. The minimum of 6 descriptive attributes that best described mango aroma characteristics were generated in the discussion session by consulting with SPME volatile profiles analysed previously. Fresh and heated purée samples of each single variety (50 mL) were served in a 100 mL plastic cup with lids [20]. The panelists accessed each attribute on a 15-cm unstructured scale to rate their intensities where the left-hand end of the scale was ‘‘attribute not perceptible” and the right-hand end was ‘‘attribute strongly perceptible” [27].

### Volatile component separation and analysis

As described therein, the purée (100 g) was homogenised with 200 mL of chilled (4–8°C) phosphate buffer (0.1 M, pH 7.0). Thereafter, phase separation was achieved by centrifugation at 5,000 rpm for 30 min (4–8°C). The supernatant was passed through a column (50 cm length x 1 cm i.d.) packed with 20 mL of AmberliteXAD-2 and method of Lalel et al. [28,29] was followed. Free volatile compounds were eluted with 100 mL of pentane/dichloromethane mixture (2:1, v/v). The fraction containing free volatiles was dried using anhydrous sodium sulphate and concentrated to dryness under vacuum at 45 ^o^C. Prior to GC-MS [2] injection, the dried extract was redissolved in 100 μL of dichloromethane comprising of toluene 0.003% (w/v) as an internal standard, the resulting solutions (1 μL) were injected onto the GC-MS system, previously described. Mass spectra was taken on the ionize mode at 70 eV from m/z 20–350 using a scanning speed of 0.5 scans/s. The n-alkane standards (C8-C20) (Fluka® Analytical, Munich, Germany) in hexane were used for the calculation of linear retention indices (LRI). The identification was done by comparison of mass spectra with those in the library (NIST 05), accordingly. The amount of the volatiles was calculated relatively to that of the internal standard [30]. The contribution of volatile compounds was also estimated using odour activity values (OAVs), which is calculated as the ratio between the concentration found in the matrix and the odour threshold of the individual active volatiles [31].

### Statistical analysis

All analyses were done using the XLSTAT version 2018. Scatter plot and box plot graphs were used to evaluate the correlation of specific gravity and mango variety. Exponential decay nonlinear regression analysis was fitted for accessing the relationship between the specific gravity and firmness at confidence level of 95%. Heat mapping of free organic volatiles was generated using an online software (http://www.heatmapper.ca). For the statistical analysis of the consumer test, the degree of liking data were subjected to the Analysis of Variance (ANOVA) followed by Duncan’s multiple range test at p < 0.05. Unless stated otherwise, the results are illustrated as mean ± SE (standard error) of triplicate analyses. The relationship between mango varieties (with heat treatment) and volatiles was analysed using principal component analysis (PCA). The volatile data were also subjected to agglomerative Hierarchical Clustering (AHC) for simply interpretation with a PREFMAP feature in the statistical package.

## Results and discussion

### Ripening stage determination

**Fig 1:**
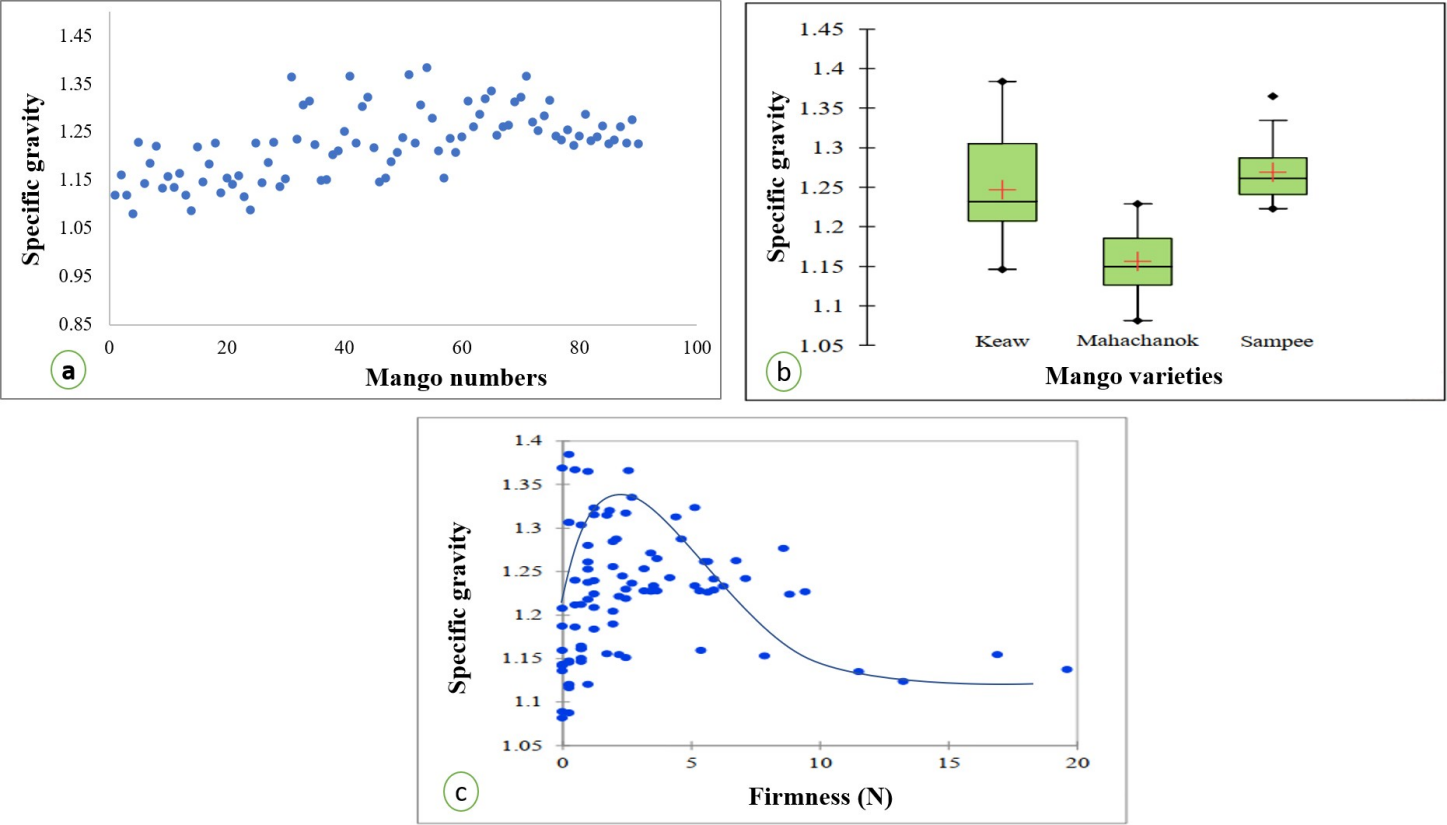
Determination of the ripening stage of Thai mango fruits by specific gravity (a) and Box plots diagram of specific gravity of different mango varieties (b) and two decay relationship of specific gravity and fruit firmness (c).

Specific gravity was used to describe fruit soluble matters and since the content of sugars increased with the improving onset of ripening, the value is generally used to define the stage of ripening [32]. In mangoes, the specific gravity of ca. 1.02 is the index for the optimum ripening stage for consumption [33]. Kalid *et al*. [21] also added that the optimum matured fruits of mango had specific gravities between 1.01 and 1.02, while the overripe fruits had the values greater than 1.02. For the Thai variety, the specific gravity of fully mature ‘nam-dok-mai’ was ~1.03 [34,35]. In this study, the mango fruit of all varieties achieved more than 1.05 of the specific gravity with the maximum value being ~1.40 (Fig 1a). Slight variations of the specific gravity have been found among different varieties (Fig 1b). Box plots illustrated that specific gravity of ‘keaw’ and ‘sam-pee’ was not significantly different while ‘maha-chanok’ gave approximately 1.15 which was different from the others. This explains the variety dependency of the specific gravity.

Another technique to elucidate fruit ripening stage is texture measurement. During the ripening stage, the firmness dropped rapidly due to alteration in cell wall structure by enzymatic degradation (e.g., polygalacturonase) and also polysaccharide degradation which led to breakdown of starch, cellulose and hemicellulose [36,37]. This is highly apparent as ripe mangoes are soft to touch [38,39]. The correlation of specific gravity and firmness was evaluated (Fig 1c). In our work the non-destructive specific gravity was well fitted in the Two decay relationship (exponential curve) with fruit firmness regardless of the varieties;

$$Specific\;gravity=‐3.22*exp\left(+0.00184*Firmness\right)+‐0.111*exp\left(‐0.742*Firmness\right)+4.453;\;R^{2}∼0.6$$

For this instance, we anticipate that the loss of firmness due to the conversion of insoluble pectin into soluble forms during the ripening stage additionally contribute to the increase in specific gravity particularly before the fruits reached the over-mature stage [33,40,41]. By using these data, we confirmed that fruits of the three varieties used in this study had reached the over-ripe stage.

The physicochemical properties of the over-ripe mango pulp (pH, titratable acid, total soluble solid, and sugar contents) of each variety were determined (Table 1). From the results, only the TSS was not significantly different among tested varieties. The pH data showed that ‘keaw’ pulp was highly acidity (pH~3.9). The value however is less than that of ‘sam-pee’ (pH~4.5) which falls within the range reported by Chambers *et al*. [42]. The determination of acid content illustrated that ‘keaw’ gave the highest, 0.9% titratable acid followed by ‘sam-pee’ and ‘maha-chanok’ 0.5–0.7%. Islam *et al*. [43] reported that fruit acidity reduced with the attainment of maturity and ripening depends upon the varieties.

**Table 1:** Physicochemical properties of over-ripe mango flesh and their sugar compositions.

	‘sam-pee’	‘maha-chanok’	‘keaw’
pH	4.5^c^ ± 0.30	4.1^ab^ ± 0.69	3.9^a^ ± 0.38
Titratable acid (%)	0.5^ab^ ± 0.21	0.7^a^ ± 0.62	0.9^a^ ± 0.37
Total soluble solid (%)^ns^	11.6 ± 1.29	10.7 ± 2.43	11.1 ± 1.06
Fructose (%)	2.19^b^ ± 0.12	3.10^a^ ± 0.15	2.22^b^ ± 0.12
Glucose (%)	2.10^a^ ± 0.11	1.62^b^ ± 0.06	1.04^d^ ± 0.05
Sucrose (%)	12.52^b^ ± 0.58	12.01^b^ ± 0.55	14.29^a^ ± 0.31

Values are mean of three measurements ± standard error (SE); Within the same row, values with the same subscription letter are not significantly different at p = 0.05.

TSS is one of the main parameters to describe maturity index as well as an indicator for mango fruit quality [33]. Although TSS content represents carbohydrates, organic acids, proteins, fats and minerals, changes in TSS during fruit ripening are mainly associated with the increase alteration of carbohydrate to simple sugars [44]. Nordey *et al*. [45] reported the TSS content increased ~2-folds during the process of ripening in mango fruits. The TSS of all mango varieties was between 10.7–11.6 Brix in which glucose, fructose and sucrose constituted the majority of carbohydrates [39]. In our work, ‘maha-chanok’ had the highest fructose content (3.10%), which is higher than those of ‘keaw’ and ‘sam-pee’ (2.19–2.22%). ‘Sam-pee’ had the highest glucose level (2.10%) and ‘keaw’ variety had the lowest glucose (1.04%). In terms of sucrose content, ‘keaw’ variety contained higher sucrose (14.29%) than that of ‘sam-pee’ and ‘maha-chanok’ varieties in which the values fall between 12.01–12.52%. These variations in sugar content may depend upon either the varieties or agronomic factors [44].

### Descriptive sensory analyses of non-heated and heated mango purée

**Fig 2:**
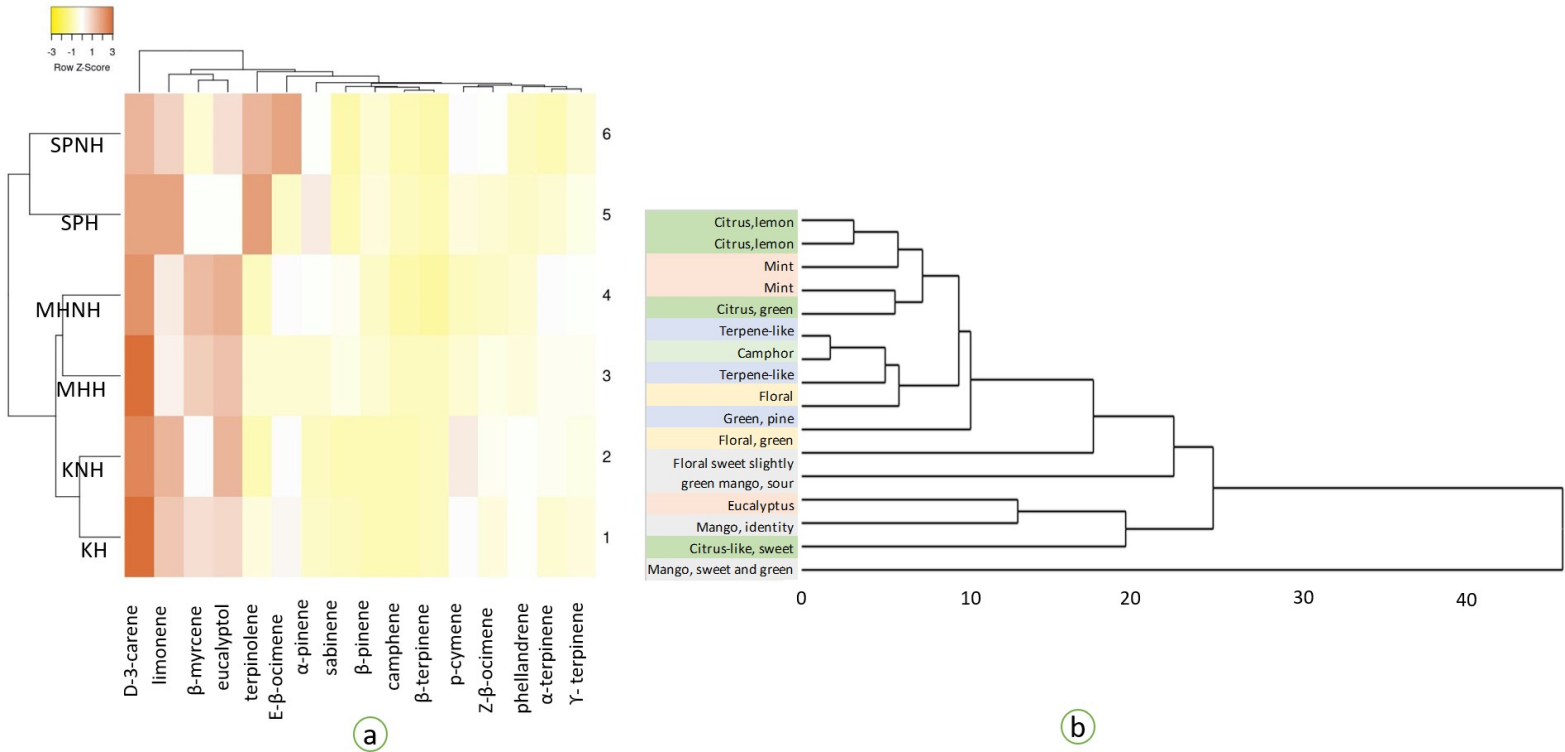
Intensity heat-mapping of the volatile compounds from solid-phase micro extraction of Thai mango purée (a) and the dendrogram of their aroma characteristics as described in Boonbumrung *et al*. [59]; Alvarez *et al*. [46] and Maldonado-Celis *et al*. [6] (b); ‘sam-pee’(SP) ‘maha-chanok’ (MH) and ‘keaw’ (K), following by H indicates sample subjected to thermal processing at 85°C for 15 minutes and NH indicates non-heat treatment.

Solid-phase microextraction (SPME) is a rapid, sensitive and non-solvent metabolomics tool for exclusive characteristics of the aromatic volatiles of fruit pulp as well as the whole fruit in food process industry [16,28]. The incubation period is usually optimised depending on the characteristics of the fruit and incubating conditions [25,28,31,46]. In this study, the mango purée of ‘sam-pee’, ‘maha-chanok’ and ‘keaw’ were incubated with the SPME fibre for 4 hr to allow the equilibrium between the fruit and head space. The SPME profiles of the volatiles emitted from the over-ripe mangoes purée before and after heat treatment are illustrated in Fig 2. These samples predominately consisted of the mixture of terpene hydrocarbons and oxygenated sesquiterpenoids including D-3-carene, limonene and β-mycene which were similar to those of different varieties reported in other studies [8,25,47]. Heat mapping in Fig 2a illustrated that the purée of ‘keaw’ and ‘maha-chanok’ were distinctive by the higher intensity of mango and citrus aroma (D-3-carene, β-mycene and limonene). The dendrogram of the aroma characteristics also illustrated that the aroma of Thai mango purée was dominantly categorised by mango identities (D-3-carene and β-mycene); citrus (limonene); sweet-floral (terpinolene; β-ocimene; sabinene) (Fig 2b). Heat treatment slightly altered the chemical compositions and their intensities but did not affect the overall aromatic profiles which were in-agreement with previous study [48].

From the result of the organic volatiles, we then nominated 5 descriptors for the later sensory analysis viz., “citrus”, “green”, “mango identity”, “tropical fruit”, and “sweet and floral” (Table 2). The sixth descriptor namely, “fermented” was also added based on previous work [49]. The ANOVA was employed to determine the differences of sensorial scoring between mango purée of the three varieties (Table 3). The statistical analysis demonstrated that each variety illustrated their own aromatic profile identities. The aroma of ‘sam-pee’ purée was intense in all descriptive categories as compared with the others. Heat treatment influenced aromatic profile by significantly improved “mango identity” in ‘maha-chanok’ purée and “fermented” odour in ‘keaw’ purée. The panelists were also able to detect a significant reduction in “green” aroma of ‘maha-chanok’ purée. The alterations of these profiles could then be used to describe the variations of each variety characteristics [50]. Suwonsichon *et al*. [51] advised that for Thai mango varieties, the aromatic attributes such as “mango identity”, various fruit notes, “floral/perfume”, “peel‐like”, “piney” and “spice” could be used to differentiate the varieties.

**Table 2:** Attributes and references used in evaluating in mango purée aromatic profile.

Aromatic attributes	Definition	Reference standards	n/15	References
Citrus	The citrus aroma associated with lemon, lime	200 mL of mango nectar mixed with 30 mL fresh squeezed lemon	10/15	Nassur *et al*. [53]
Green	The greenish aroma associated with unripe fruits	Slices of granny smith apple	7/15	Ledeker *et al*. [20]
Mango identity	A sweet, fruity, green, somewhat woody and piney aromatic associated with mango that sometimes may include aromatics similar to other specific fruits, such as peach, orange, grapefruit and/or pineapple	200 mL of mango nectar	6.5/15	Ledeker *et al*. [20]
Tropical fruit	A sweet, woody, slightly sharp, floral aromatic associated with pineapple	Diluted Dole canned pineapple juice with water (1:1)	6/15	Ledeker *et al*. [20]
Fermented	Over-ripe tropical fruit or apple cider	Mango juice with 30% Heinz vinegar	10/15	Chambers *et al*. [42]

Aroma perception is generally specified by a diverse range and intensity of volatile compounds synthesised through physiological changes of the fruits [52,53]. For ‘ataulfo’ mango, “tropical fruit” and “peach” aromas increased to the optimum at the full ripening stage of the fruit, which was opposite to the variety ‘haden’ [53]. For Thai mango varieties such as ‘nam-dok-mai’, ‘keaw’ and ‘chok-anan’, the intensities of “fermented”, “green” and “tropical fruit” aromas were reduced at their full ripening stage [51]. Pre-processing may also increase the sensorial intensity of mango. The flavour intensities “mango identity” and “tropical fruit” significantly increased in ‘chok-anan’ as in the form of purée while decreased the “green” and “pine” flavours [20]. The “fermented” and “sweet and floral” aromas of Thai mango purée remained as fresh fruits in ‘chok-anan’, ‘keaw’, ‘nam-dok-mai’, ‘nung-klang-won’, ‘ok-rong’ and ‘tong-dum’ [20]. In our previous screening of Thai mango aroma, we also confirmed that heat treatment alternated the intensity of sensory descriptors and the sweet and floral scent were intensively increased in ‘keaw’, ‘maha-chanok’, ‘chok-anan’ and ‘sam-pee’. The green and citrus aromas, however, were reduced [54].

**Table 3:** Descriptive sensory analysis of Thai mango purée.

Descriptive characteristics	‘sam-pee’		‘maha-chanok’		‘keaw’	
	NH	H	NH	H	NH	H
**Citrus**	2.18^b^	2.55^b^	1.09^b^	2.18^b^	5.55^a^	3.82^ab^
**Green**	5.09^ab^	5.23^ab^	3.29^ab^	1.41^c^	3.68^bc^	4.00^bc^
**Mango Identity**	5.82^abc^	7.05^a^	3.27^d^	5.73^abc^	4.68^bcd^	5.91^ab^
**Tropical Fruit**	7.55^a^	7.95^a^	3.36^c^	5.36^abc^	4.27^bc^	6.27^ab^
**Fermented**	2.02^bc^	3.18^ab^	0.59^c^	1.36^c^	1.91^bc^	3.91^a^
**Sweet and floral**	4.55^ab^	4.18^ab^	3.64^ab^	3.59^ab^	3.45^ab^	5.18^a^

^*^Same subscription letters indicate no statistical difference (*P* ≥ 0.05) between means according to Duncan’s test; H indicates sample subjected to thermal processing at 85°C for 15 minutes and NH indicates non-heat treatment.

### Odour activity value of free volatile components

**Fig 3:**
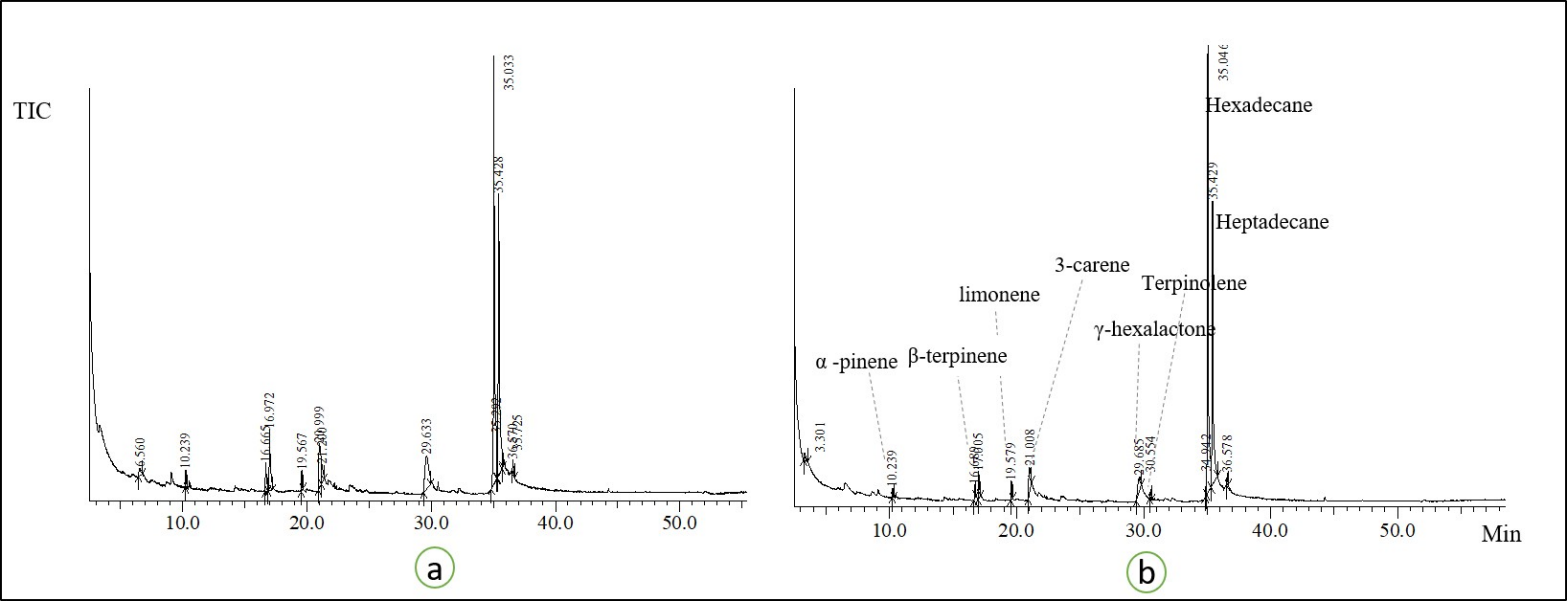
Chromatograms of free volatiles analysed from purée; ‘sam-pee’(SP) subjected to heat treatment (a) and non-heat treatments (b).

**Fig 4:**
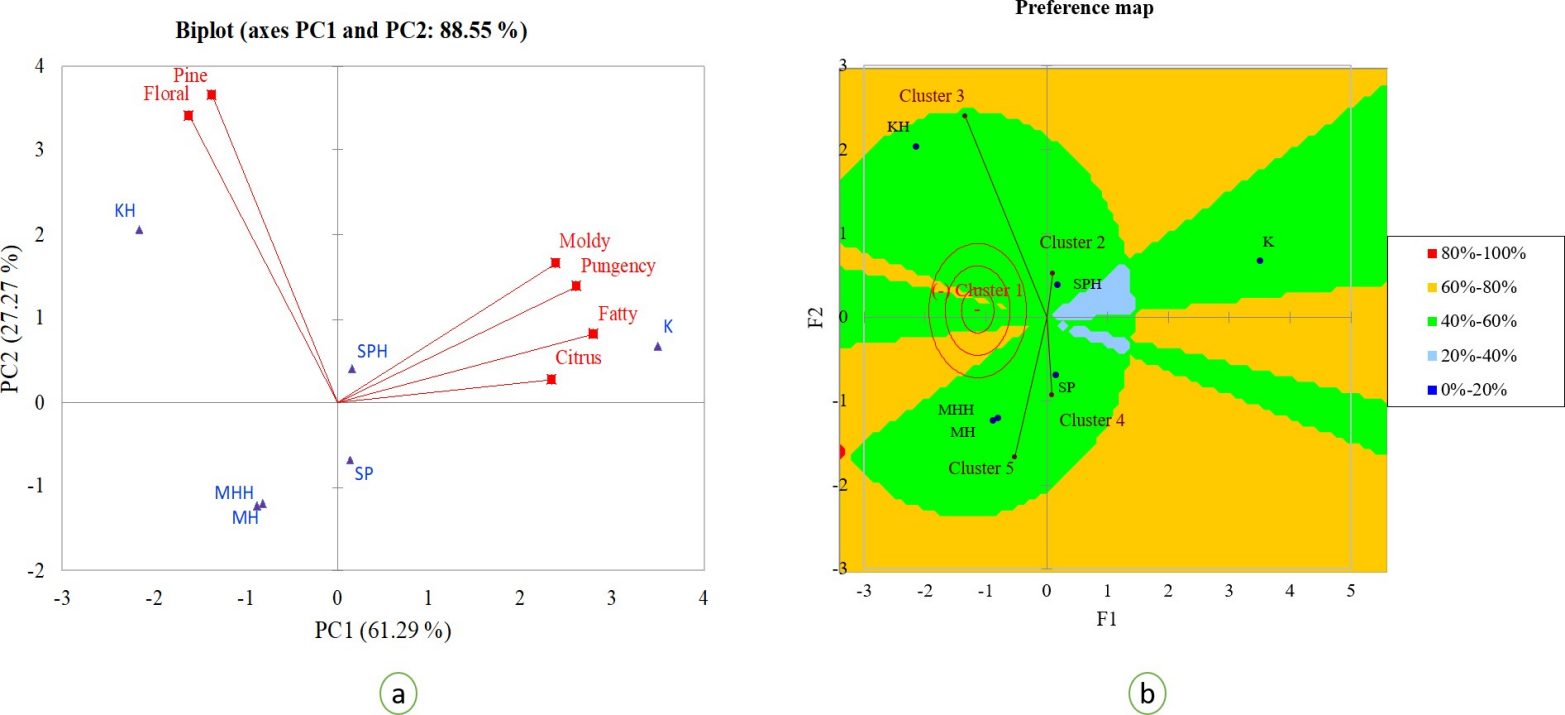
PCA biplot illustrating the relationships of the volatile descriptors and the volatile components (a) and the circular ideal point model on PREFMAP of OAVs of the volatiles from mango purée; ‘sam-pee’(SP) ‘maha-chanok’ (MH) and ‘keaw’ (K), following by H indicates sample subjected to thermal processing at 85°C for 15 minutes and NH indicates non-heat treatment.

For the calculation of odour activity value (OAV), odour thresholds of the volatiles from mango fruits of different varieties were used as reported by Pino and Mesa [55] along with the additional values reported from other fruits and beverages [9,56,57]. The OAVs usually describe the threshold concentration of such compounds present in the fruit, which determines the contribution of the volatiles to the overall odour profile of the fruit [55]. The amounts of free volatiles were quantified from the chromatograms and used to calculate OAVs. Fig 3a and 3b show the chromatograms of the detected volatiles from heat and non-heat sam-pee mango. From the analyses, 19 components were detected from free volatile fraction of Thai mango purée as listed in Table 4. The citrus representing compounds such as β-terpinene, γ- terpinene and limonene were principal in all varieties, with 2,5-dimethyl-4-methoxy-3(2H)-furanone of the nutty sweet aroma standing out (OAV > 1700) in ‘keaw’ purée. Monoterpene hydrocarbons such as δ-3-carene, limonene, terpinolene, β-pinene and γ-terpinene are the typical chemotypes for the identity descriptions of mango varieties in previous studies [8,9,31,54,55]. To determine the factual effect of heat treatment, the volatile components within the same descriptors were gathered into the same class. The multidimensional relationship among the samples, based on their profile characteristic of the aroma, was displayed on the PCA score plot. The first two dimensions of the PCA accounted for 61.29% (PC1) and 27.27% (PC2) of the variance (Fig 4a). The PCA-biplot with profile descriptor illustrated that heat treatment played a significant effect on the aroma profile of ‘keaw’ purée by altering the intensities of the chemical volatiles representing pine and floral aroma. The treatment also modified the onset of the odour profile in ‘sam-pee’ mango. Again, the analysis showed that the scent of mango purée from ‘maha-chanok’ was heat resistant. Moreover, in order to make the interpretation of results easier, we then grouped the OAVs of the 19 detected components into 5 homogeneous groups according to AHC clustering groups (S1 Dataset). The PREFMAP was then generated using the coordinates of the type of mango purée in the two-dimensional factor space and the OAVs that had been categorised into 5 clusters using a circular ideal point model. The result showed that with or without heat treatment, the aromatic profile of ‘maha-chanok’ filled in the same cluster with ~50% of the volatile correspondents (Fig 4b). In this same figure, the contour map illustrated ‘keaw’, ‘maha-chanok’ and ‘sam-pee’ purée possessed a higher proportion of models with the intensity of profile characteristics (accounted for 40–60%). Heat treatment did have an apparent impact on the volatile profiles of ‘keaw’ and ‘sam-pee’ as the samples were split into different clustering groups. In different studies, the treatment could generally affect the volatiles compositions through chemical degradations of terpene hydrocarbons such as those representing peel-like and pine attributes [20,58]. Moreover, the variations in sweet-floral aroma could be as the results of acetyl furan and 5-methyl furfural formation via ascorbic acid degradation [20] and Maillard reaction [59] which are not the focus in this study.

**Table 4:** Chemical compositions of the extracted free volatiles of non-heated and heated mango purée prepared from over-ripe mango fruits.

	Compounds	Formula	LRI	Odour	Descriptors	SP		SPH		MH		MHH		K		KH	
				threshold (ppb)		ug/g	OAV	ug/g	OAV	ug/g	OAV	ug/g	OAV	ug/g	OAV	ug/g	OAV
**1**	α-thujene	C_10_H_16_	921	22	Green, herb, pungency	nd		nd		nd		nd		0.30±0.01	13.73	nd	
**2**	α -pinene	C_10_H_16_	927	6	Green, pine	nd		0.034±0.00075	5.71	nd		nd		nd		0.022±0.0029	3.69
**3**	β-terpinene	C_10_H_16_	968	2	Terpene-like	0.029 ±0.002	14.5	0.015±0.000225	7.39	0.0058±0.00025	2.88	0.0089±0.00015	4.43	nd		0.030±0.00095	15.02
**4**	α-terpinolene	C_10_H_16_	975	200	Floral sweet slightly green mango, sour			0.37±0.035	1.83	nd		nd		nd		nd	
**5**	decane	C_10_H_22_	998	100	Citrus-like, sweet	0.010 ±0.0035	0.1	nd		nd		nd		nd		nd	
**6**	α-terpinene	C_10_H_16_	1015	1000	Citrus,lemon	nd		nd		nd		nd		nd		nd	
**7**	limonene	C_10_H_16_	1026	10	Citrus-like, sweet	0.033 ±0.001	3.3	0.12±0.1	12	0.0049±0.0001	0.49	0.0075±0.00054	0.75	0.30±0.015895	30.51	0.029±0.0033	2.93
**8**	D-3-carene	C_10_H_16_	1049	770	Mango, sweet and green	0.0085 ±0.0005		nd		nd		nd		0.10±0.00298	0.13	0.030±0	0.039
**9**	Z-ocimene	C_10_H_16_	1051	6.7	Mint	nd		nd		nd		nd		nd		nd	
**10**	γ-hexalactone	C_6_H_10_O_2_	1056	50	Coumarin, sweet	0.013 ±0.001	0.26	0.011±0.00185	0.22	nd		nd		nd		0.062±0.0029	1.24
**11**	2,5-dimethyl-4-methoxy-3(2H)-furanone	C_7_H_10_O_3_	1062	0.03	Nutty sweet	nd		nd		nd		nd		0.052±0.0037	1743.33	0.0079±5.5E-05	264.83
**12**	Terpinolene	C_10_H_16_	1088	140	Floral sweet slightly green mango, sour	nd		0.028± 0.005	0.2	nd		nd		nd		1.28±0.03	9.14
**13**	1-phenyl-2-butene	C_10_H_12_	1094	nd	nd	nd		nd		nd		nd		nd		0.50±0.0097	
**14**	γ -terpinene	C_10_H_16_	1179	2	Citrus	0.022 ±0.005	11	nd		nd		nd				nd	
**15**	dodecane	C_12_H_26_	1201	nd	nd	0.030 ±0.005		0.032± 0.0032		0.0056 ±0.00042		0.018 ±0.0056		0.03±0.0014		nd	
**16**	tetradecane	C_14_H_30_	1401	nd	nd	0.038± 0.004		0.060± 0.0014		0.0089 ±0.00012		0.028 ±0.0037		nd		0.019±0.00052	
**17**	hexadecane	C_16_H_34_	1601	nd	Alkane	0.25±0.22		0.053±0		0.0046± 0.00044		0.023± 0.0037		0.040±0.0024		0.014±0.0012	
**18**	heptadecane	C_17_H_36_	1801	nd	Alkane	0.018± 0.0015		0.03±0		nd		0.016±0.0050		nd		nd	
**19**	ethyl hexadecanoate	C_18_H_36_O_2_	1901	2000	Fruity, sweet	nd		nd		nd		0.0069±0.0001	0.0034	nd		nd	

‘sam-pee’(SP) ‘maha-chanok’ (MH) and ‘keaw’ (K), following by H indicates sample subjected to thermal processing at 85°C for 15 minutes and NH indicates non-heat treatment.

## Conclusions

In summary, heat processing altered the intensity of aromatic sensorial profiles of mango purée from 3 Thai varieties with high market need for processing. The analysis of free volatiles by GC-MS illustrated 19 volatile components leading with those representing mango identity such as δ-3-carene, β-mycene and limonene. The aroma profiles of Thai mango purée were also categorised based on the citrus and sweet-floral characteristics which were unique for Thai variety. The PCA with PREFMAP elucidated that the aromatic profiles could distinguish these varieties and heat treatment had largely influenced the profile in the purée of ‘keaw’, while ‘sam-pee’ and ‘maha-chanok’ could remain their aroma identity. The finding can be advantageous to fruit processing industry whose interest is to preserve the aroma identity of mango varieties through product and development process.

## Supporting information

S1 Dataset The statistical data for Table 4. (PDF)
