# Supplementary material for: Volatile profiles from over-ripe purée of Thai mango varieties and their physiochemical properties during heat processing
Source: PLoS One. 2021 Mar 17;16(3):e0248657. doi: 10.1371/journal.pone.0248657 (PMC7968669; doi:10.1371/journal.pone.0248657)
Supplement: S1 Dataset — (PDF) [file pone.0248657.s001.pdf]

You are using the XLSTAT trial version. Number of days remaining until the trial expire

XLSTAT 2020.2.3.65348 - Agglomerative hierarchical clustering (AHC) - Start tim
Observations/variables table: Workbook = Preference mapping.xlsx / Sheet = PF
Row labels: Workbook = Preference mapping.xlsx / Sheet = PREFERENCE map /
Cluster rows
Dissimilarity: Euclidean distance
Agglomeration method: Ward's method
Center: Yes
Reduce: Yes
Truncation: number of classes: 5

Summary statistics

Summary statistics:

Variable	Observations	. with missing	vithout missin	Minimum	Maximum
SP	23	0	23	0.000	14.500
SPH	23	0	23	0.000	12.000
MH	23	0	23	0.000	2.875
MHH	23	0	23	0.000	0.006
K	23	0	23	0.000	1743.333
KH	23	0	23	0.000	264.833

Node statistics:

Node	Level	Weight	Objects	Left son	Right son
45	33.328	23	23	33	44
44	22.885	17	17	39	43
43	13.283	12	12	40	42
42	9.333	8	8	25	41
41	6.667	6	6	24	28
40	2.532	4	4	3	38
39	1.281	5	5	36	37
38	0.196	3	3	10	34
37	0.180	2	2	7	13
36	0.166	3	3	8	35
35	0.058	2	2	1	11
34	0.001	2	2	12	14
33	0.000	6	6	23	32
32	0.000	5	5	22	31
31	0.000	4	4	21	30
30	0.000	3	3	20	29
29	0.000	2	2	18	19
28	0.000	4	4	16	27

27	0.000	3	3	15	26
26	0.000	2	2	6	9
25	0.000	2	2	5	17
24	0.000	2	2	2	4

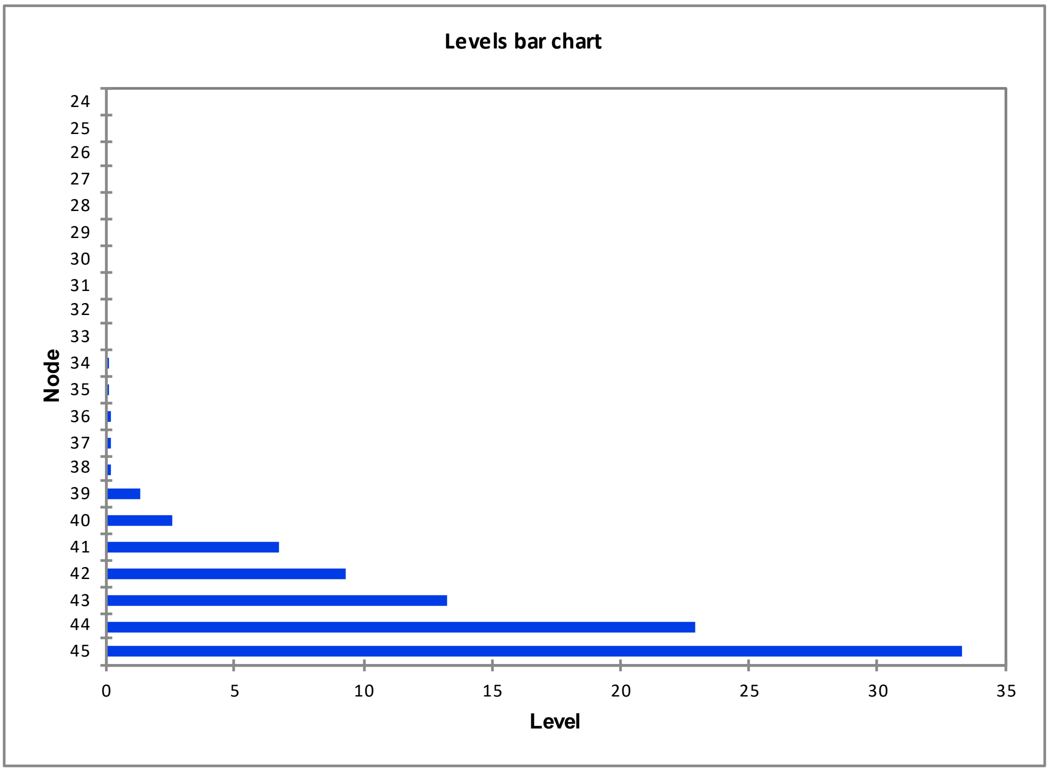

Cophenetic distance matrix:

	1	2	3	4	5
1	0.000	22.885	22.885	22.885	22.885
2	22.885	0.000	13.283	6.667	9.333
3	22.885	13.283	0.000	13.283	13.283
4	22.885	6.667	13.283	0.000	9.333
5	22.885	9.333	13.283	9.333	0.000
6	22.885	6.667	13.283	6.667	9.333
7	1.281	22.885	22.885	22.885	22.885
8	0.166	22.885	22.885	22.885	22.885
9	22.885	6.667	13.283	6.667	9.333
10	22.885	13.283	2.532	13.283	13.283
11	0.058	22.885	22.885	22.885	22.885
12	22.885	13.283	2.532	13.283	13.283
13	1.281	22.885	22.885	22.885	22.885
14	22.885	13.283	2.532	13.283	13.283
15	22.885	6.667	13.283	6.667	9.333
16	22.885	6.667	13.283	6.667	9.333
17	22.885	9.333	13.283	9.333	9.333
18	33.328	33.328	33.328	33.328	33.328

19	33.328	33.328	33.328	33.328	33.328
20	33.328	33.328	33.328	33.328	33.328
21	33.328	33.328	33.328	33.328	33.328
22	33.328	33.328	33.328	33.328	33.328
23	33.328	33.328	33.328	33.328	33.328

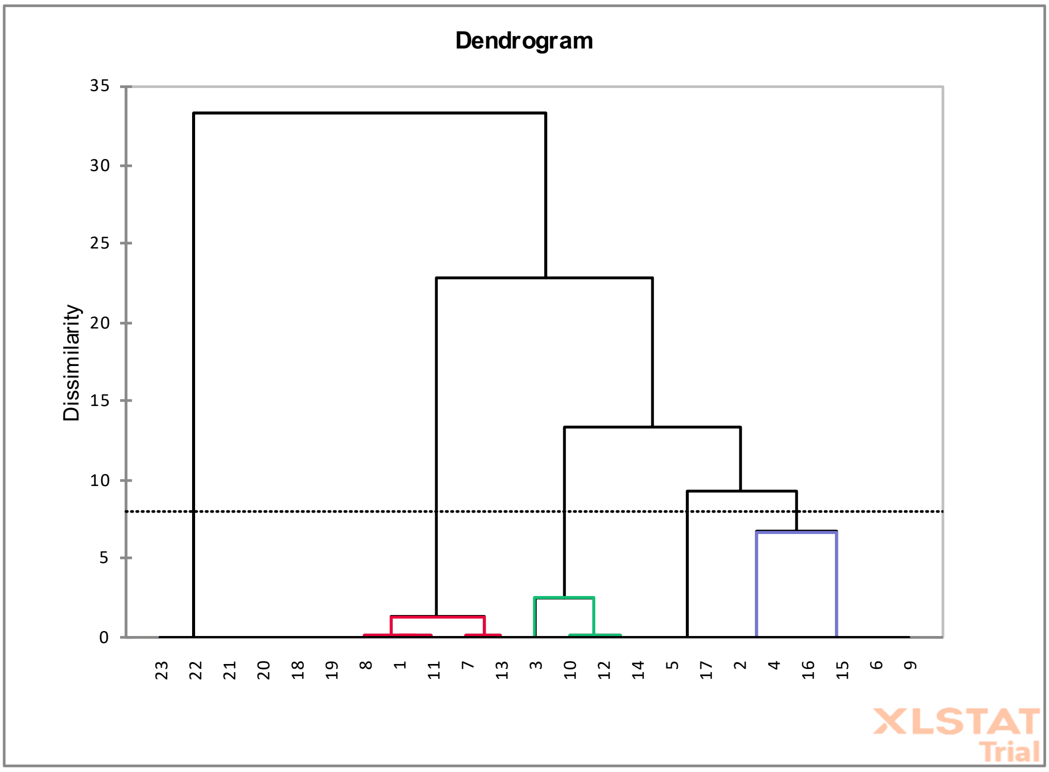

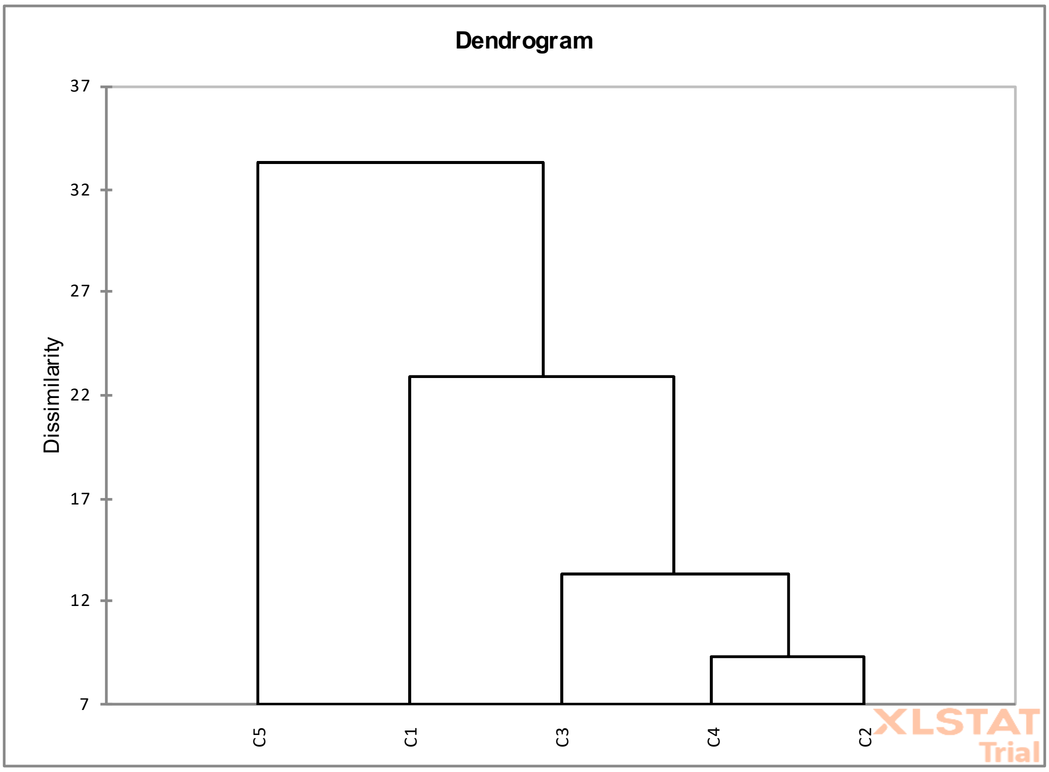

Variance decomposition for the optimal classification:

	Absolute
Within-class	136303.287
Between-classes	
Total	134894.659

Class centroids:

Class	SP	SPH	MH	MHH	K
Cluster 1	1.016	2.883	0.231	0.000	358.670
Cluster 2	0.000	1.256	0.000	0.000	0.000
Cluster 3	3.690	1.953	0.719	0.000	0.000
Cluster 4	5.553	0.000	0.000	0.000	0.000
Cluster 5	0.000	0.000	0.000	0.003	0.000

Distances between the class centroids:

	1	2	3	4	5
1	0	362.653	361.656	362.687	362.660
2	362.653	0	8.216	5.693	1.256
3	361.656	8.216	0	7.790	8.416
4	362.687	5.693	7.790	0	5.553
5	362.660	1.256	8.416	5.553	0

Central objects:

Class	SP	SPH	MH	MHH	K
1 (7)	3.300	12.000	0.490	0.001	30.511
2 (4)	0.000	1.830	0.000	0.000	0.000
3 (14)	0.000	0.200	0.000	0.000	0.000
4 (5)	0.105	0.000	0.000	0.000	0.000
5 (19)	0.000	0.000	0.000	0.004	0.000

Distances between the central objects:

	1 (7)	2 (4)	3 (14)	4 (5)	5 (19)
1 (7)	0	32.466	33.463	33.075	33.085
2 (4)	32.466	0	9.280	1.833	1.830
3 (14)	33.463	9.280	0	9.139	9.138
4 (5)	33.075	1.833	9.139	0	0.105
5 (19)	33.085	1.830	9.138	0.105	0

Results by class:

Class	1	2	3	4	5
Objects	5	6	4	2	6
Sum of weig	5	6	4	2	6
Within-class	613264.776	5.293	104.751	59.351	0.000
Minimum dis	332.176	0.574	4.547	5.448	0.001
Average dista	560.309	1.675	7.953	5.448	0.002
Maximum di	1400.692	4.452	14.531	5.448	0.003
	1	2	3	5	18
	7	4	10	17	19
	8	6	12		20
	11	9	14		21
	13	15			22
		16			23

Results by object:

Observation	Class
1	1
2	2
3	3
4	2
5	4
6	2
7	1
8	1
9	2
10	3
11	1
12	3
13	1
14	3
15	2
16	2
17	4
18	5
19	5
20	5
21	5
22	5
23	5

es: 13

ne: 2020-05-27 at 19:05:58 / End time: 2020-05-27 at 19:05:59

REFERENCE map / Range = 'PREFERENCE map'!$C$2:$H$25 / 23 rows and 6 columns

' Range = 'PREFERENCE map'!$A$2:$A$25 / 23 rows and 1 column

Mean	Std. deviation
1.345	3.716
1.294	3.029
0.175	0.612
0.001	0.002
77.972	363.101
12.908	55.036

6	7	8	9	10	11	12
22.885	1.281	0.166	22.885	22.885	0.058	22.885
6.667	22.885	22.885	6.667	13.283	22.885	13.283
13.283	22.885	22.885	13.283	2.532	22.885	2.532
6.667	22.885	22.885	6.667	13.283	22.885	13.283
9.333	22.885	22.885	9.333	13.283	22.885	13.283
0.000	22.885	22.885	6.667	13.283	22.885	13.283
22.885	0.000	1.281	22.885	22.885	1.281	22.885
22.885	1.281	0.000	22.885	22.885	0.166	22.885
6.667	22.885	22.885	0.000	13.283	22.885	13.283
13.283	22.885	22.885	13.283	0.000	22.885	0.196
22.885	1.281	0.166	22.885	22.885	0.000	22.885
13.283	22.885	22.885	13.283	0.196	22.885	0.000
22.885	0.180	1.281	22.885	22.885	1.281	22.885
13.283	22.885	22.885	13.283	0.196	22.885	0.001
6.667	22.885	22.885	6.667	13.283	22.885	13.283
6.667	22.885	22.885	6.667	13.283	22.885	13.283
9.333	22.885	22.885	9.333	13.283	22.885	13.283
33.328	33.328	33.328	33.328	33.328	33.328	33.328

33.328	33.328	33.328	33.328	33.328	33.328	33.328
33.328	33.328	33.328	33.328	33.328	33.328	33.328
33.328	33.328	33.328	33.328	33.328	33.328	33.328
33.328	33.328	33.328	33.328	33.328	33.328	33.328
33.328	33.328	33.328	33.328	33.328	33.328	33.328

KH
53.560
0.000
7.273
0.000
0.000

KH
2.930
0.000
9.136
0.000
0.000

13	14	15	16	17	18	19
1.281	22.885	22.885	22.885	22.885	33.328	33.328
22.885	13.283	6.667	6.667	9.333	33.328	33.328
22.885	2.532	13.283	13.283	13.283	33.328	33.328
22.885	13.283	6.667	6.667	9.333	33.328	33.328
22.885	13.283	9.333	9.333	9.333	33.328	33.328
22.885	13.283	6.667	6.667	9.333	33.328	33.328
0.180	22.885	22.885	22.885	22.885	33.328	33.328
1.281	22.885	22.885	22.885	22.885	33.328	33.328
22.885	13.283	6.667	6.667	9.333	33.328	33.328
22.885	0.196	13.283	13.283	13.283	33.328	33.328
1.281	22.885	22.885	22.885	22.885	33.328	33.328
22.885	0.001	13.283	13.283	13.283	33.328	33.328
0.000	22.885	22.885	22.885	22.885	33.328	33.328
22.885	0.000	13.283	13.283	13.283	33.328	33.328
22.885	13.283	0.000	6.667	9.333	33.328	33.328
22.885	13.283	6.667	0.000	9.333	33.328	33.328
22.885	13.283	9.333	9.333	0.000	33.328	33.328
33.328	33.328	33.328	33.328	33.328	0.000	33.328

33.328	33.328	33.328	33.328	33.328	33.328	0.000
33.328	33.328	33.328	33.328	33.328	33.328	33.328
33.328	33.328	33.328	33.328	33.328	33.328	33.328
33.328	33.328	33.328	33.328	33.328	33.328	33.328
33.328	33.328	33.328	33.328	33.328	33.328	33.328

20	21	22	23
33.328	33.328	33.328	33.328
33.328	33.328	33.328	33.328
33.328	33.328	33.328	33.328
33.328	33.328	33.328	33.328
33.328	33.328	33.328	33.328
33.328	33.328	33.328	33.328
33.328	33.328	33.328	33.328
33.328	33.328	33.328	33.328
33.328	33.328	33.328	33.328
33.328	33.328	33.328	33.328
33.328	33.328	33.328	33.328
33.328	33.328	33.328	33.328
33.328	33.328	33.328	33.328
33.328	33.328	33.328	33.328
33.328	33.328	33.328	33.328
33.328	33.328	33.328	33.328
33.328	33.328	33.328	33.328
33.328	33.328	33.328	33.328

33.328	33.328	33.328	33.328
0.000	33.328	33.328	33.328
33.328	0.000	33.328	33.328
33.328	33.328	0.000	33.328
33.328	33.328	33.328	0.000
